# Sex Differences in the Study of Neurological Illnesses

**DOI:** 10.1155/2015/676531

**Published:** 2015-12-29

**Authors:** Hrayr Attarian, Jan Brandes, Rima Dafer, Elizabeth Gerard, Barbara Giesser

**Affiliations:** ^1^Northwestern University Feinberg School of Medicine, Chicago, IL 60611, USA; ^2^Nashville Neuroscience Group, Nashville, TN 37203, USA; ^3^Northshore University HealthSystem, Glenview, IL 60026, USA; ^4^UCLA, David Geffen School of Medicine, Los Angeles, CA 90095, USA

There are clear sex based neurophysiological differences in brain structure and function. These impact both healthy individuals and those with neurological and psychiatric disorders. It is well documented that these diseases affect women differently from men. Many of these sex differences remain unknown and underrecognized. In addition, hormonal changes and fluctuations during a woman's lifespan are significantly more numerous and more complex than in men. These hormonal changes can impact the pathogenesis and the clinical presentation of neurological illness as well as a woman's response to treatment. There are definite sex and gender differences in prevalence of various neurological illnesses, in the incidence of psychiatric comorbidities, and in the therapeutic responses to various pharmacological and nonpharmacological interventions.

For instance, Alzheimer's disease (AD), Parkinson's disease (PD), multiple sclerosis (MS), and other neurodegenerative illnesses have different prevalence and distinct presentation in women compared to men. Depression, both on its own and as a comorbid condition with neurological illnesses, has a gender specific presentation, impact, and therapeutic response. Antiepileptic drugs and other neurological medications can interact with hormonal contraception causing unplanned pregnancies. Hormonal contraception can impact risk of stroke, and migraine headaches fluctuate throughout the menstrual period. There are reports of MS symptoms fluctuating in response to different phases of the menstrual cycle. Sleep disorders increase in pregnancy and menopause and can affect the health of mother and fetus in the former and significantly reduce quality of life in the latter. There are also complex issues of managing specific disorders such as migraines, epilepsy, restless legs syndrome, and multiple sclerosis (MS) in pregnancy. Last but not least stroke, the third cause of death worldwide, has very unique sex and gender based symptomatology and semiology.

Despite these distinctions, there is a dearth of research in specific aspects of neurology as it relates to women's health. Often in clinical trials the data is not specifically separated by gender.

We invited authors to submit original research, case series, case reports, or review papers that address this disparity of research in women and look into neurologic and behavioral sex based changes in healthy individuals, the specific pathophysiology of neuropsychiatric illnesses in women, epidemiological and health based social disparities, and the differential effect of therapeutic interventions in women.

After rigorous peer review we decided to include eight papers in this special issue. These cover gender differences in neurodegenerative diseases like AD and PD as well as sex specific interface of cognitive function and mood disorders. We also included articles on gender specific burden of MS care, sex differences in childhood neuroborreliosis, and behavioral symptoms in Prader-Willi syndrome. Lastly two intriguing contributions were also accepted for publication and these are on the role of estrogen deficiency in subarachnoid hemorrhage and the role of gender in sociosexuality of infidelity after traumatic brain injury.

In summary this special edition of this journal includes a wide range of papers addressing the sex and gender differences in neuropsychiatric pathophysiology and care. We hope it will increase awareness of these sex and gender based variations in the presentation of neurologic illness and care in the medical community.

*Hrayr Attarian* *Jan Brandes* *Rima Dafer* *Elizabeth Gerard* *Barbara Giesser*

